# Autoantibody and biomarker detection in the follow-up of autoimmune diseases: state of the art and future perspectives

**DOI:** 10.1016/j.jtauto.2025.100346

**Published:** 2025-12-20

**Authors:** Jan Damoiseaux, Yves Renaudineau

**Affiliations:** a Central Diagnostic Laboratory, Maastricht University Medical Center, Maastricht, the Netherlands; b Immunology Department Laboratory, Referral Medical Biology Laboratory, Institut Fédératif de Biologie, University Hospital of Toulouse, INSERM, U1297, Toulouse, France

**Keywords:** Autoimmune diseases, Monitoring, Disease activity, Flares, Therapy response

## Abstract

Originally developed for the diagnosis of autoimmune diseases, the application of autoantibodies and related biomarkers has been extended in certain cases to monitor therapeutic efficacy and to predict disease recurrence and/or severity. Several critical considerations must be addressed prior to employing autoantibodies for monitoring purposes: (i) whether the autoantibody is pathogenic, as exemplified by anti-glomerular basement membrane and anti-acetylcholine receptor autoantibodies; (ii) whether the autoantibody level is essential for tracking disease activity, which may necessitate establishing prognostic thresholds and/or adapting detection methodologies; (iii) whether there is added value in combining autoantibodies, as demonstrated with anti-dsDNA and anti-chromatin antibodies in systemic lupus erythematosus; (iv) which immunoglobulin isotype is optimal for monitoring; and (v) whether alternative biomarkers exist that provide greater accuracy for patient follow-up. Additional issues remain unresolved, including appropriate intervals between measurements, intra- and inter-laboratory reproducibility, inter-assay variations, and ethnic variability, among others. To address these challenges, the European Autoimmunity Standardization Initiative (EASI) has proposed adapted strategies for utilizing autoantibodies in the longitudinal assessment of selected autoimmune diseases, as presented in this special issue.

## 1 Introduction

Autoantibodies, being either pathogenic or only bystander, are well recognized as diagnostic markers for autoimmune diseases. Their role in the follow-up of autoimmune diseases, that is repeated analyses over time, is more diverse. In the current special issue of the Journal of Translational Autoimmunity, entitled “Autoantibodies and immunological markers in the follow-up of autoimmune diseases”, initiated by the European Autoimmunity Standardization Initiative (EASI) [1], the added value of autoantibody monitoring, either on its own or in combination with other biomarkers, is addressed in the context of distinct autoimmune diseases. For some autoantibodies such a role is not evident at all as reported with extractable nuclear antigens and anti-citrullinated protein/peptide antibodies [2,3], while other autoantibodies such as anti-dsDNA and anti-tissue transglutaminase have been used to monitor treatment efficacy by achieving clinical remission, to monitor disease activity over time, or to predict recurrent disease activity [4,5]. Below we will elaborate on these applications with some typical examples, but also pinpoint important gaps in our knowledge as related to the laboratory assays involved (Table 1). Although autoantibodies measured at the time of diagnosis might be prognostic for disease progression over time, this will not be discussed here, but is differentially included in the contributions to this special issue.

**Table 1:** Autoantibodies and biomarkers for follow-up in autoimmune diseases (selected examples).

Disease Biomarker	Remission induction	Disease activity	Prediction relapse	Remarks^a^
SLE^b^				• Anti-C1q and usCD163 are markers for lupus nephritis • C3 is decreased during active disease, but may be counter-balanced as being an acute phase protein • Biomarkers for remission induction and disease activity should be interpreted as changes over time in individual patient by taking into account biological variation and inter-assay variation • Follow-up measurements of autoantibodies should be performed with the same assay • Definition of increase in autoantibody levels to predict relapse is ill-defined
Anti-dsDNA	++	++	+	
Anti-Chromatin	++	++	ND	
Anti-C1q (LN)	++	++	+	
Anti-Sm	–	–	ND	
Anti-SSA	–	–	ND	
C3	+	+	ND	
C4	+	+	ND	
usCD163 (LN)	++	++	ND	
Systemic sclerosis				• There is no evident role for autoantibodies in monitoring systemic sclerosis • Autoantibodies to the so-called extractable nuclear antigens generally do not change over time in qualitative terms (negative – positive); quantitative results are usually not reported
Anti-CENP-B	–	–	–	
Anti-Topoisomerase I	–	–	–	
Goodpasture's disease				• Achieving a sero-negative status for anti-GBM upon plasmapheresis depends on the cut-off of the chosen assay • Goodpasture's disease does not typically relapse, making monitoring or autoantibodies for disease activity and prediction of relapse redundant
Anti-GBM	++	NA	NA	
ANCA-associated vasculitis				• Remission induction and disease activity are primarily based on a clinical score including organ involvement (BVAS) • usCD163 is a biomarker for renal involvement, i.e., glomerulonephritis • PR3-ANCA patients are more prone to relapse and prediction is best in patients with renal involvement
MPO-ANCA	+	+	ND	
PR3-ANCA	+	+	+	
usCD163	++	++	ND	
Rheumatoid arthritis				• There is no evident role for autoantibodies in monitoring in RA • Monitoring for IgM-RF might be relevant in patients with type II cryoglobulinemia, irrespective of a diagnosis RA
IgM-RF	–	–	–	
ACPA	–	–	–	
Myasthenia gravis				• For autoantibodies to AChR, in contrast to antibodies to MuSK, a ∼50 % reduction seems sufficient for achieving a clinical relapse
Anti-AChR	++	+	ND	
Anti-MuSK	++	++	ND	
NMDA-R encephalitis				• Relapses are primarily based on clinical parameters, but a seroconversion may support a relapse
Anti-NMDA-R	++	+	ND	
Multiple sclerosis				• There is no evident role for immunoglobulin intrathecal production in monitoring multiple sclerosis
OCBs	+/−	ND	ND	
Kappa index	+/−	ND	ND	
Autoimmune gastritis				• APCA, and to a lesser extend anti-IF, are insufficient to establish ongoing autoimmune gastritis on its own, but in combination with functional gastric biomarkers the are considered of added value
APCA	–	+	ND	
Anti-IF	–	+/−	ND	
PGI/PGII	–	+	ND	
G17	–	+	ND	
Celiac disease				• Achieving a sero-negative status for anti-tTG upon GFD depends on the cut-off of the chosen assay • In patients with an IgA deficiency, IgG anti-tTG is less responsive to a GFD • While i-FABP indicates gastro-intestinal damage, GIP defines recent gluten intake
IgA anti-tTG	++	+	ND	
i-FABP	+	+	ND	
GIP	–	+	ND	

a General remarks (last three bullet points) made for biomarkers in SLE also account for the other diseases.

b Abbreviations: AChR, acetyl-choline receptor; ACPA, anti-citrullinated protein antibodies; ANCA, anti-neutrophil cytoplasmic antibodies; APCA, anti-parietal cell antibodies; BVAS, Birmingham vasculitis activity score, dsDNA, double stranded deoxyribonucleic acid; G17, gastrin 17; GFD, gluten free diet; GIP, gluten immunogenic peptides; IF, intrinsic factor; i-FABP, intestinal fatty acid binding protein; LN, lupus nephritis; MPO, myeloperoxidase; MuSK, muscle-specific kinase; NA, not applicable; ND, not determined, NMDA-R, N-methyl-D-aspartate receptor; OCBs, oligoclonal bands; PGI/II, pepsinogen I/II; PR3, proteinase 3; RA, rheumatoid arthritis; RF, rheumatoid factor; tTG, tissue transglutaminase.

## 2 Autoantibody utility to predict therapy response

Monitoring treatment efficacy is most evident for those autoantibodies that are well established to be pathogenic. Autoantibodies to the glomerular basement membrane (GBM) in Goodpasture's disease are typical examples. The disease is characterized by an acute onset and requires fast analysis for the presence of anti-GBM antibodies in the circulation and/or deposition in the glomeruli. Treatment includes multiple rounds of plasmapheresis to remove the autoantibodies not only from the circulation, but also out of the tissues. It is helpful to use an assay that reveals quantitative results in order to evaluate treatment efficacy. Plasmapheresis is to be continued until the anti-GBM assay reveals negative results. It can be questioned, however, if the cut-off differentially defined for diagnostic purposes is also adequate for the decision to stop plasmapheresis [6]. Another example of pathogenic autoantibodies includes the anti-acetyl-choline receptor (AChR) antibodies in myasthenia gravis (MG). While the pathogenicity of these autoantibodies is undisputable, the intended target of reduction in autoantibody levels is considered to be about 50 % of the original level at the time of diagnosis. A patient with high levels of anti-AChR antibodies, therefore, is considered to be in clinical remission while still a substantial amount of autoantibodies is present. This can be explained by the differential rapsyn-based interaction of the AChR in the neuromuscular endplate with the cytoskeleton of the muscle cell. Strong interactions with the cytoskeleton require high amounts of anti-AChR antibodies to cause muscle weakness [7]. For the anti-AChR antibody assays it is detrimental that quantitative results are obtained in an assay with sufficient linearity. The latter may be at stake in the for diagnostic purposes considered gold standard radio-immuno-assay (RIA). The measuring range is rather small and requires multiple dilutions to obtain a quantitative value. Linearity of autoantibody assays upon dilutions is most often limited due to the increasing participation of low-affinity autoantibodies in higher dilutions. Besides these examples of pathogenic autoantibodies, monitoring of IgA anti-tissue transglutaminase (tTG) antibodies in celiac disease illustrates that also bystander autoantibodies might be applied. Both efficacy of as well as strict adherence to a gluten-free diet can be monitored by the presence or absence of IgA anti-tTG autoantibodies, with biomarkers like intestinal free acid binding protein (i-FABP) and gluten immunogenic peptides (GIP) of potential added value. As indicated for the pathogenic antibodies, there is added value for quantitative results and the cut-off defined by diagnostic companies for diagnostic purposes may not be appropriate for follow-up purposes [5].

## 3 Autoantibody monitoring to predict relapses

The use of autoantibodies to predict clinical relapses raises some additional challenges. Increases in the level of anti-dsDNA antibodies and anti-neutrophil cytoplasmic antibodies (ANCA) have been reported to predict renewed disease activity in, respectively, systemic lupus erythematosus (SLE) and ANCA-associated vasculitis (AAV). The major challenge is the definition of degree of increase, time between consecutive blood draws, time between increase and relapse, clinical characteristics of the relapse, and the type of assay to be performed. The original study that is most often referred to in order to link anti-dsDNA antibody levels with an SLE relapse was based on a limited number of SLE patients (n = 34), with a well-defined definition of the antibody rise (>25 %, >15 IU, and within 4 months), and based on immuno-assays (in-house ELISA and commercial Farr-assay) that are currently not available anymore [8]. Similarly, the original study to relate rises in ANCA levels with an AAV relapse was based on only 35 C-ANCA positive AAV patients. ANCA levels were measured every month by indirect immunofluorescence (IIF) on ethanol-fixed neutrophils, a rise was defined by two-titer steps (4 times increase), and a relapse was differentiated in minor and major relapses [9]. In this respect, it should be noted that since the revision of the international consensus on ANCA detection in 2017 the autoantibodies are predominantly quantified in antigen-specific immuno-assays instead of IIF [10]. It remains to be determined how a rise in autoantibody level, either anti-dsDNA antibodies or ANCA, is to be defined and interpreted. It is evident that also for this application the cut-off provided by the diagnostic companies is of no added value for the intended purpose of predicting a clinical relapse. Since both SLE and AAV are heterogeneous diseases, it is also to be determined if prediction is valid for all patients or only a subset of patients, for instance those with renal involvement, as suggested by Kemna et al. in AAV [11]. An additional challenge is whether prediction can be improved by combining the autoantibody results with other types of biomarkers.

## 4 Concluding remarks, challenges and opportunities

Overall, it is evident that for some autoantibodies the added value of follow-up measurements is disputed, while for other autoantibodies there is good support. In this context it is relevant to realize that assays for autoantibodies have been introduced into the market only for diagnostic purposes and not for follow-up. The extended use of such assays raises questions about the cut-off value to be used, this will be different for diagnostic and follow-up purposes, and about the correct interpretation of changes in quantitative values. For the latter it is important to realize that autoantibody assays are not standardized and are most often not linear upon dilution of the serum. For this application it is mandatory to measure the consecutive samples in the same immuno-assay, preferentially simultaneously in the same dilution(s). Moreover, perhaps other autoantibody characteristics, like avidity and glycosylation, might be relevant for the correct interpretation. Importantly, the non-intended use of autoantibody assays beyond the diagnostic purpose is in conflict with the European IVD-R 2017/746 and implicates that laboratories have to take additional measures to enable clinical monitoring. A close collaboration between clinicians, laboratory specialists, and the diagnostic companies is required to fill these gaps for full application of autoantibodies, either or not in combination with other biomarkers, in the follow-up of autoimmune diseases.

## CRediT authorship contribution statement

**Jan Damoiseaux:** Conceptualization, Writing – original draft, Writing – review & editing. **Yves Renaudineau:** Conceptualization, Writing – review & editing.

## Funding

This research did not receive any specific grant from funding agencies in the public, commercial, or not-for-profit sectors.

## Declaration of competing interest

The authors declare that they have no known competing financial interests or personal relationships that could have appeared to influence the work reported in this paper.

## Data Availability

No data was used for the research described in the article.
